# Antenatal and neonatal exposure to SARS-CoV-2 and children’s development: a systematic review and meta-analysis

**DOI:** 10.1038/s41390-023-02954-y

**Published:** 2023-12-19

**Authors:** Rebecca Jackson, Kathryn Woodward, Meg Ireland, Conor Larkin, Jennifer J. Kurinczuk, Marian Knight, Chris Gale, Samantha Johnson, Rosie Cornish, Ela Chakkarapani

**Affiliations:** 1 Translational Health Sciences, Bristol Medical School, University of Bristol, Bristol, United Kingdom; 2 National Perinatal Epidemiology Unit, Nuffield Department of Population Health, University of Oxford, Oxford, United Kingdom; 3 Neonatal Medicine, School of Public Health, Imperial College London, London, United Kingdom; 4 Department of Population Health Sciences, University of Leicester, Leicester, United Kingdom; 5 Population Health Sciences, Bristol Medical School, University of Bristol, Bristol, United Kingdom

## Abstract

**Objectives:**

To conduct a systematic review of the impact of antenatal and neonatal exposure to SARS-CoV-2 on developmental outcomes in preterm and term-born infants.

**Methods:**

We searched Embase, Emcare, MEDLINE, PsycINFO, Web of Science and grey literature on May 27, 2022 and updated on May 8, 2023. Studies defining exposure with a positive SARS-CoV-2 protein or genetic material, used a contemporaneous non-exposed cohort, and reported developmental outcomes up to 2 years of age were included.

**Results:**

Four out of 828 screened studies were included. Meta-analysis included 815 infants screened for developmental delay (*n* = 306 exposed; *n* = 509 non-exposed) between 3- and 11-months of age. Among term-born infants, we did not find an increased risk of delay in communication (odd’s ratio: 0.73 (95% CI: 0.24–2.24)), gross motor (1.50 (0.62, 3.62)), fine motor (2.90 (0.58, 14.43)), problem-solving (1.19 (0.54, 2.66)) or personal-social development (1.93 (0.78, 4.75)) in exposed infants. The number of preterm-born infants in the exposed (*n* = 37) and comparison cohorts (*n* = 41) were too few to report meaningful comparisons.

**Conclusion:**

Evidence regarding the potential impact of antenatal or neonatal exposure to SARS-CoV-2 infection on developmental outcomes in early infancy is limited and inconsistent. Larger cohorts with outcomes beyond the first year of life are needed.

**Impact:**

- The current evidence examining associations between SARS-CoV-2 exposure during the neonatal period and developmental outcomes in infancy is limited by there being few studies with extremely small sample sizes.
- Based on sparse data there was no consistent association between antenatal or neonatal exposure to SARS-CoV-2 infection and an adverse impact on developmental outcomes below 12 months of age for babies born preterm or at term.
- This study highlights that larger cohorts with outcomes assessed beyond the first year are needed to determine the potential longer-term impact of SARS-CoV-2 infection exposure on child development.

## Introduction

As of June 2023, it is estimated that SARS-CoV-2 virus has infected over 767 million people worldwide, and over 22 million people in the UK alone.^1,2^ Pregnant women who contract SARS-CoV-2 infection are at increased risk of developing severe COVID-19 disease. During the global pandemic, compared with uninfected pregnant women, pregnant women with SARS-CoV-2 infection had a significantly higher risk of mortality, admission to intensive care, receipt of mechanical ventilation or any critical care, and being diagnosed with pneumonia or thromboembolic disease.^3–6^ Consequently, infants born to mothers with SARS-CoV-2 infection were more likely to be born preterm (<37 weeks of gestation) or moderate preterm (<34 weeks of gestation), low birth weight (<1500 g) or admitted to a neonatal unit than those born to uninfected mothers;^3^ of the infants admitted to a neonatal unit, 67% were preterm and 68% received respiratory support.^7,8^ SARS-CoV-2 infection may also have adverse long-term effects on infant development: exposure to SARS-CoV-2 during pregnancy or in the neonatal period may impact foetal or neonatal brain development either directly through central nervous system infection, indirectly through the adverse effects of viral infection related inflammation in the mother or infant, or secondary to preterm birth or other consequences of maternal COVID-19.^9–15^

A number of cohort studies have examined the impact of antenatal and/or neonatal exposure to SARS-CoV-2 on infant development at different ages and in different countries. To date, there are three systematic reviews examining the impact of maternal SARS-CoV-2 infection on neurodevelopmental outcomes.^16–18^ All three reviews reported an increased risk of neurodevelopmental impairment in children exposed to SARS-CoV-2 during pregnancy compared with a non-exposed cohort to 12 months of age. However, these previous systematic reviews did not investigate outcomes separately for preterm and term-born infants. Given the high risk of preterm birth following maternal SARS-CoV-2 infection, and increased risk of adverse neurodevelopmental outcomes following preterm birth^19^ it is essential to understand whether the risk of neurodevelopmental impairment differs depending upon whether SARS-CoV-2 infants were born preterm or at term. Moreover, there are currently no data on the neurodevelopmental outcomes of infants who had SARS-CoV-2 infection in the neonatal period. Therefore, the aim of this study was to conduct a systematic review of the impact of antenatal and neonatal exposure to SARS-CoV-2 infection on developmental outcomes in preterm and term-born infants.

## Methods

This systematic review was conducted in accordance with the PRISMA guidelines, and was registered in PROSPERO (CRD42022314063, http://www.crd.york.ac.uk/PROSPERO/).

### Inclusion and exclusion criteria

We searched for observational studies, including cohort and case–control studies, which examined the impact of SARS-CoV-2 exposure on child development for 1) children of mothers who tested positive for SARS-CoV-2 during pregnancy (any of the three trimesters) and 2) children who tested positive for SARS-CoV-2 in the neonatal period (i.e., the first 28 days after birth). SARS-CoV-2 exposure was determined by detection of coronavirus protein (e.g., lateral flow test) or detection of coronavirus genetic material (e.g., polymerase chain reaction, PCR). Only studies which included a contemporaneous comparison group of non-exposed infants were included in the review. A non-exposed comparison group was defined as a group without evidence of exposure to SARS-CoV-2 infection during pregnancy or in the neonatal period.

### Outcome measures

The primary outcome was development in childhood assessed using a validated measure, including in the domains of cognition, communication, social-emotional, motor, sensory (including hearing) and global development. The children’s age at the time of the outcome assessment was limited to ≤2 years as the COVID-19 pandemic was declared by the World Health Organisation in March 2020.

### Data sources and search strategy

**Fig. 1:**
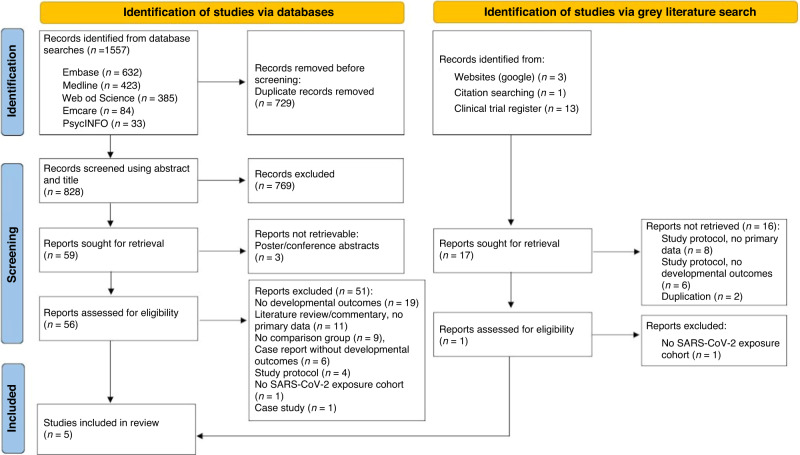
Study flow chart. Flow chart prepared according to the PRISMA guidelines template for meta-analyses.

We conducted an initial extensive search of the following databases: Embase, Emcare, MEDLINE, PsycINFO and Web of Science, which were searched between the 11^th^ and 27^th^ of May 2022. The grey literature was also searched between the 6th of July and 8th of August 2022, which included the World Health Organisation website, clinical trials.gov, Open Grey, Google Scholar, and the references cited in the eligible articles. Subsequent searches were conducted between the 23^rd^ of November and 16^th^ of December 2022 and the 1^st^ and 8^th^ of May 2023 to screen for additional articles. We restricted our search to articles published between 2020 and 2023. No language restrictions were applied. We excluded conference abstracts, case-studies, and letters or opinions without any clinical data. Supplementary Table S1 supplementary materials shows the search terms used and Fig. 1 illustrates the PRISMA flow chart.^20^

### Study selection process

Following identification of articles, duplicates were removed (Fig. 1). The titles and/or abstracts of articles retrieved were independently screened for relevance based on our inclusion/exclusion criteria by a minimum of three investigators (KW, RJ, EC, MI, and CL). The full text of potentially eligible articles was retrieved and independently assessed for eligibility by three investigators (RJ, MI, and CL). Any discrepancies between the investigators or uncertainties over eligibility were resolved through discussion with a fourth investigator (KW).

### Data collection process

Data were extracted from each article and independently entered in Excel data extraction sheets by three investigators (RJ, MI, and CL). The extracted data were then cross verified by KW, who checked and resolved any discrepancies or uncertainties.

Extracted study population details included cohort type, sample size, details on how exposure was confirmed (e.g., positive PCR), when exposure occurred, and severity of the exposure (e.g., hospitalisation). We also extracted adequacy of follow-up of cohorts, methods used to analyse the data, and any confounders which were adjusted for. Extracted outcome data included details of the type of outcome assessed, the measure(s) used, estimates of the effect (e.g., mean difference or odds ratio), the age of the children at assessment, and term (≥37 weeks’ gestation) and preterm (<37 weeks’ gestation) birth status of the exposed and non-exposed infants. We approached the study authors to obtain outcome data by preterm or term gestation at birth for four articles included in the meta-analysis^21–24^ where this was not reported in the publication. The data we requested were the mean scores with standard deviations as well as proportion of infants with developmental delay stratified by exposure status and term and preterm birth status.

### Risk of bias assessment

The quality of the studies was assessed using the Newcastle–Ottawa Scale (NOS). Three investigators (RJ, MI and CL) performed the risk of bias assessment independently, and disagreements were resolved through a discussion with KW.

### Synthesis of results

A text-based summary of study results was undertaken, whereby studies were grouped according to the type of outcome measure used, and sub-grouped according to age of assessment and exposure type (antenatal or neonatal), and whether the infant was preterm or term-born. For each developmental domain, pooled effect estimates comparing exposed and non-exposed infants (odds ratio (OR) for developmental delay and standardised mean difference (SMD) for test scores) and their 95% confidence intervals were obtained using a random effects meta-analysis with inverse variance weighting. This was done separately for term and preterm infants. Heterogeneity was assessed using I^2^ statistics.

Results were summarised narratively for one study examining developmental outcomes from SARS-CoV-2 infection exposure in the neonatal period.

### Missing data

Two of the studies^21,22^ did not provide complete information needed to carry out the meta-analysis. In one study, the total numbers with missing ASQ-3 data in the exposed and non-exposed groups were provided in the article but we did not receive these numbers stratified by term/preterm status. In another study, the article reported that five exposed infants and one non-exposed infant had fine motor delay; the article reported details, including gestational age, for the five exposed infants but not the non-exposed one. To carry out the meta-analysis we made assumptions about these missing data; we carried out sensitivity analyses with different assumptions. Further details are provided in the Supplementary Materials.

## Results

### Study selection and characteristics

A total of 1557 articles were identified via database searches; 828 articles remained after de-duplication. Following title and abstract screening, 59 articles were sought for retrieval and 56 articles were assessed for eligibility. Seventeen articles were further identified via grey literature searches, and two of these articles were assessed for eligibility. Eligible articles identified via database and grey literature searches, a total of five articles were included in the final review (Fig. 1). Articles included in the review received risk of bias assessment scores ranging between 7 and 9 (Table 1). The characteristics of the included studies are reported in Table 2.

**Table 1:** Newcastle–Ottawa Scale assessment for risk of bias in observational studies.

Authors	Selection	Comparability	Outcomes
Firestein 2023	⋆⋆⋆⋆	⋆⋆	⋆⋆⋆
Liu 2022	⋆⋆⋆	⋆	⋆⋆⋆
Shuffrey 2022	⋆⋆⋆⋆	⋆⋆	⋆⋆⋆
Wu 2021	⋆⋆⋆	⋆⋆	⋆⋆⋆

A study can be awarded a maximum of one star for each numbered item within the Selection and Exposure categories. A maximum of two stars can be given for Comparability. Total NOS scores categorised into three groups: very high risk of bias (0–3 NOS points), high risk of bias (4–6), and low risk of bias (7–9).

**Table 2:** Characteristics of the studies included in the systematic review.

First Author (date)	Study type	Study population (sample size)	Confirmation of exposure	Exposure timing	Measure of neurodevelopment	Measure of effect	Results	Age of assessment
Firestein (2023)	Cohort	In utero exposure to maternal SARS-CoV-2 infection cohort (*n* = 112: term = 95, preterm = 17) Non-exposed cohort (*n* = 258: term = 239, preterm = 19)	Nasopharyngeal specimen PCR and serological testing for SARS-CoV-2 antibodies	1st trimester (*n* = 14) 2nd trimester (*n* = 41) 3rd trimester (*n* = 36) Note: exact timing of exposure could be determined for 91 of 112 (81%)	Developmental Assessment of Young Children, 2nd edition (DAYC-2)	Mean differences in each DAYC-2 subdomain: cognitive, gross motor, fine motor, expressive language, and receptive language development.	No difference between the exposed and non-exposed cohort for cognitive, gross motor, fine motor, expressive language, or receptive language development using DAYC-2 subdomain scores.	~5–11 months
Liu (2022)	Cohort	In utero exposure to maternal SARS-CoV-2 infection cohort (*n* = 28: term = 23, preterm = 5) Non-exposed cohort (*n* = 48: term = 41, preterm = 7)	Positive RT-PCR	3rd trimester (*n* = 76 [100%])	Denver Developmental Screening Test, 2nd Editions (DDST-2)	Proportion of children at risk of developmental delay for each DDST-2 sub-domain: fine motor-adaptive, gross motor, personal-social, and language skills.	Developmental delay for the ‘fine-motor adaptive’ subdomain was significantly higher in the exposure cohort (15.2%) in comparison to the non-exposed cohort (2.1%). Developmental delay was not identified in the exposed cohorts for gross motor, personal-social, or language.	~5 months
Shuffrey (2022)	Cohort	In utero exposure to maternal SARS-CoV-2 infection cohort (*n* = 114: term = 107, preterm = 7) Non-exposed cohort (*n* = 141: term = 131, preterm = 10) Pre-pandemic cohort (*n* = 62)	Nasopharyngeal PCR	1st trimester (*n* = 25 [22%]) 2nd trimester (*n* = 54 [47%]) 3rd trimester (*n* = 35 [31%])	Ages and Stages Questionnaire, 3rd Edition (ASQ-3)	Mean differences and proportion at risk of developmental delays in each ASQ-3 domain: communication, gross motor, fine motor, problem solving, and personal social.	No difference between the two pandemic cohorts for the ASQ-3 in any domain. Infants in the pandemic cohort had significantly lower mean scores than historical cohort in the following ASQ-3 domains: gross motor, fine motor and personal-social.	~6 months
Wu (2021)	Cohort	In utero exposure to maternal SARS-CoV-2 infection cohort (*n* = 52: term = 45, preterm = 7) Non- exposed cohort (*n* = 62: term = 58, preterm = 4)	Positive result on high-throughput sequencing or nasopharyngeal RT-PCR	2nd trimester (*n* = 4 [7%]) 3rd trimester (*n* = 53 [93%])	ASQ-3 and Ages and Stages Social-Emotional Questionnaire, 2^nd^ Edition (ASQ:SE-2)	Proportion at risk of developmental delay for the ASQ-3 and ASQ-SE total score and in each domain.	ASQ-3 domains for gross motor, fine motor, problem solving, and personal–social scores were significantly lower in the exposure cohort. Risk of social-emotional development delay (ASQ-SE) did not differ between exposed and non-exposed cohort.	~3 months
Yan (2021)	Case-control	Neonatal SARS-CoV-2 infection cohort (*n* = 5: term = 4, preterm = 1) Non-exposed cohort (*n* = 15: term = 14, preterm = 1)	Positive RT-PCR	At birth	ASQ-3 and Hammersmith neonatal neurologic examination (HNNE)	ASQ-3 total and domain scores reported for each child in the exposure cohort only. Median differences in HNNE total and domain scores between infants with and without neonatal exposure to SARS-CoV-2 infection.	None of the exposure cohort were at risk of development delay for the ASQ-3 total score or in each ASQ-3 domain. The exposure cohort had significantly lower scores for median reflex, orientation, behaviour, and total scores on the HNNE.	~9 months

We identified a total of 897 infants in the included studies. Of these, 835 infants were included in the systematic review (62 non-exposed infants had developmental outcomes that were assessed prior to the pandemic and thus were excluded from the analysis). Three hundred and six infants (~37%) had antenatal exposure to maternal SARS-CoV-2 infection, five (<1%) infants had neonatal exposure, and 524 (~63%) had no evidence of either neonatal or antenatal exposure. A total of 757 (91%) infants were born at term and 78 (9%) were preterm. Outcomes were assessed when the children were aged between 0 and 11 months. Three studies were conducted in China,^21,22,25^ and two studies in USA.^23,24^

### Outcome assessments

Of the five studies included in the final review, three assessed development using the Ages and Stages Questionnaire, 3^rd^ Edition (ASQ-3), one used the Denver Developmental Screening Test, 2^nd^ Edition (DDST-2), and one used Developmental Assessment of Young Children, 2^nd^ Edition (DAYC-2).

### Meta-analysis

#### Developmental test scores

There was no evidence of a difference between antenatally exposed and non-exposed infants in developmental test scores in any developmental domain, for either term-born or preterm infants.

**Fig. 2:**
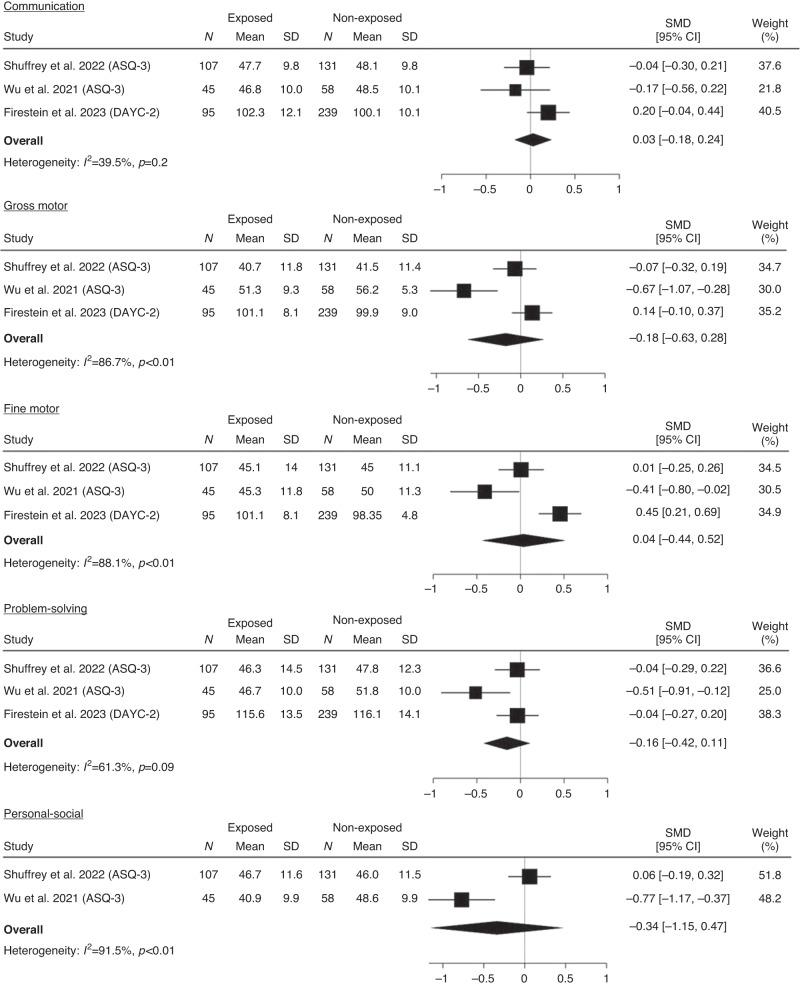
Meta-analysis of developmental test scores for communication, gross motor, fine motor, problem solving, and personal social development for exposed and non-exposed term-born infants. Note. standard mean difference (SMD), 95% confidence interval (95% CI).

Term-born infants: communication (SMD 0.03, 95% CI −0.18 to 0.24), gross motor (SMD −0.18, 95% CI −0.63 to 0.28), fine motor (SMD 0.04, CI% −0.44 to 0.52), problem solving (SMD −0.16, 95% CI −0.42 to 0.11), and personal social skills (SMD −0.34, 95% CI −1.15 to 0.47) (Fig. 2). High heterogeneity between studies was observed for the fine motor (${I}^{2}$ = 88.1%, *p* < 0.01), gross motor (${I}^{2}$ = 86.7%, *p* < 0.01), and personal social (${I}^{2}$ = 91.5%, *p* < 0.01) subdomains.

**Fig. 3:**
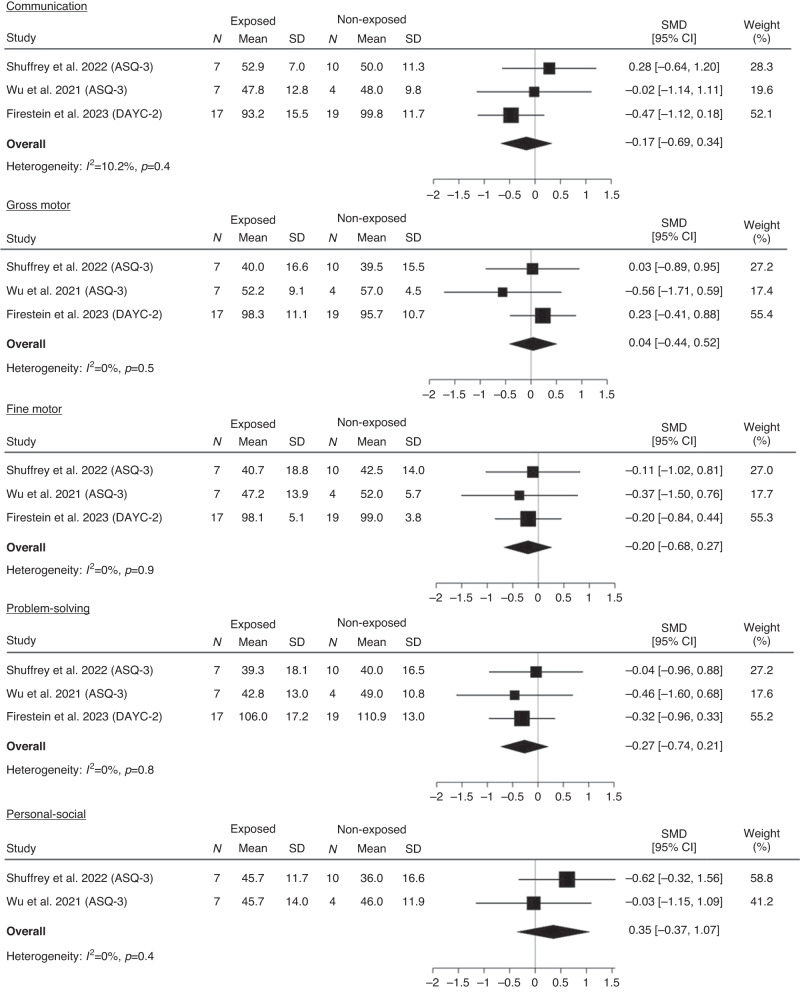
Meta-analysis of developmental test scores for communication, gross motor, fine motor, problem solving and personal social development for exposed and non-exposed preterm-born infants. Note. standard mean difference (SMD), 95% confidence interval (95% CI).

Preterm born infants: communication (SMD −0.19, 95% CI −0.67 to 0.29), gross motor (SMD 0.04, 95% CI −0.44 to 0.52), fine motor (SMD −0.20, 95% CI −0.68 to 0.27), problem solving (SMD −0.27, 95% CI −0.74 to 0.21), and personal social skills (SMD 0.35, 95% CI −0.37 to 1.07) (Fig. 3). There was low heterogeneity between studies for all developmental domains.

The results from the sensitivity analyses making different assumptions regarding missing data are given in Supplementary Table 2; there were no material differences in the findings.

#### Risk of developmental delay

**Fig. 4:**
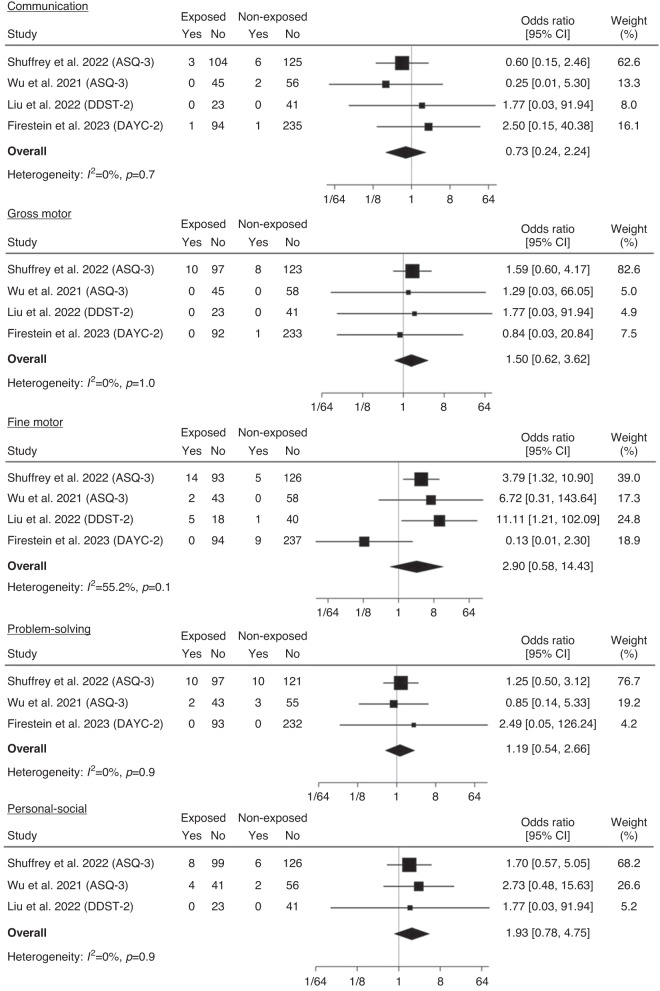
Meta-analysis of proportion at risk for delayed communication, gross motor, fine motor, problem solving, and personal social development for exposed and non-exposed term-born infants. Note. odds ratio (OR), 95% confidence interval (95% CI).

For term-born infants, there was no evidence of a difference between exposed and non-exposed infants in the risk of developmental delay in any domain: communication (OR 0.73, 95% CI 0.24–2.24), gross motor (OR 1.50, 95% CI 0.62–3.62), fine motor (OR 2.90, 95% CI 0.58–14.43), problem solving (OR 1.19, 95% CI 0.54–2.66), and personal social skills (OR 1.93, 95% CI 0.78–4.75) (Fig. 4). There was moderate heterogeneity between studies for fine motor delay (${I}^{2}$ = 55.2%, *p* = 0.1).

**Fig. 5:**
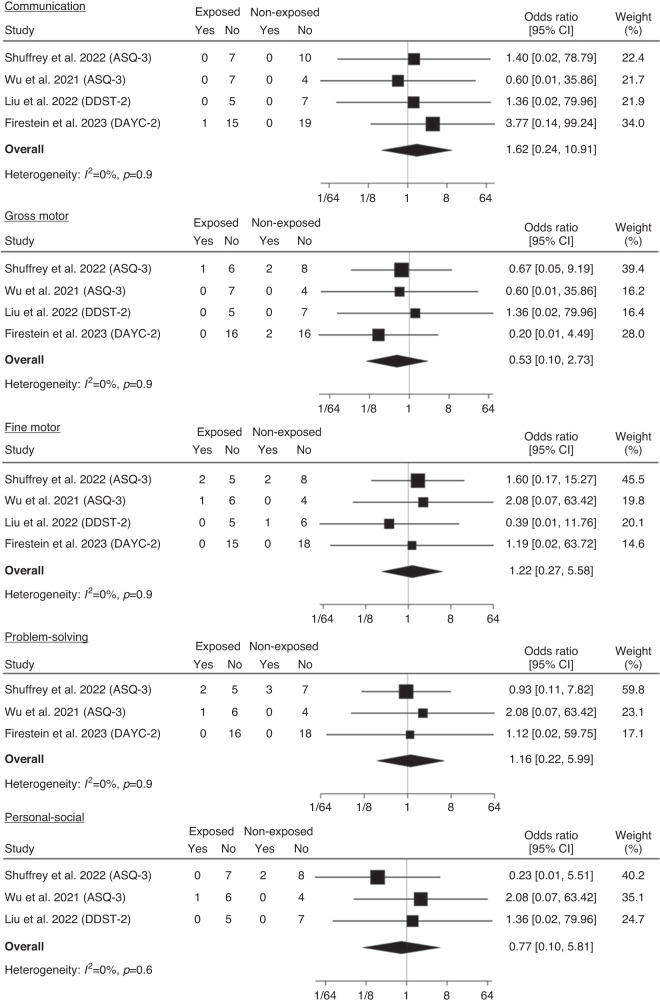
Meta-analysis of proportion at risk for delayed communication, gross motor, fine motor, problem solving and personal social development for exposed and non-exposed preterm-born infants. Note. odds ratio (OR), 95% confidence interval (95% CI).

For preterm born infants, no differences were identified between exposed and non-exposed infants for the risk of developmental delay in any domain: communication (OR 1.62, 95% CI 0.24–10.91), gross motor (OR 0.53, 95% CI 0.10–2.73), fine motor (OR 1.22, 95% CI 0.27–5.58), problem solving (OR 1.16, 95% CI 0.22–5.99) and personal social skills (OR 0.77, 95% CI 0.10–5.81 (Fig. 5). Heterogeneity between studies was low for all comparisons. Making different assumptions about missing data had no material impact on the pooled results (Supplementary Table 2).

### Exposure to SARS-CoV-2 in the neonatal period

The five neonates exposed to SARS-CoV-2 in the neonatal period scored on average 2 points lower for reflexes and 2.5 points lower for orientation and behaviour measured using the Hammersmith Neonatal Neurological Examination (HNNE). A difference was also identified for the HNNE total scores such that exposed neonates scored lower overall than non-exposed neonates. No differences were observed for other neurobehavioural items including posture and tone, tone patterns, movements, and abnormal signs/patterns. In the follow-up ASQ-3 screening of the exposed group at 9 months of age, the communication, gross motor, fine motor, problem solving, and personal social development scores for all infants were in the normal range. Data were not assessed considering preterm or term-born status (1 preterm exposed neonate and 1 control).

## Discussion

In this systematic review we summarise current evidence examining whether antenatal or neonatal exposure to SARS-CoV-2 infection is associated with poorer neurodevelopment outcomes in infancy. Building on the findings from prior systematic reviews, we investigated whether neurodevelopmental outcomes following antenatal or neonatal exposure to SARS-CoV-2 infection differed for preterm and term-born infants.

### Antenatal exposure

The results of our meta-analysis provide no evidence that, in term-born infants, antenatal exposure to maternal SARS-CoV-2 infection impacts infants’ cognitive development, communication, gross motor, problem solving, or personal social skills between the ages of 3 and 11 months of age. Similarly, in preterm born infants, we found no evidence that developmental outcomes differed between exposed and non-exposed groups. However, the preterm sample size was small and further studies with larger samples are needed to determine the impact of SARS-CoV-2 exposure on developmental outcomes in infants born preterm.

When considered independently, the findings between studies varied considerably. One study,^21^ identified lower scores for term-born exposed infants at 3-months of age for all developmental domains, while another^23^ found a small decrease in scores for problem solving, communication and gross motor development at 6 months of age. A third study^24^ identified lower scores for problem solving between 5 and 11 months of age. Variations in timing of exposure and exposure severity, as well the age at outcome assessment, may explain the disparate findings. For example, two studies^21,22^ reported findings for infants with exposure mostly in the third trimester (>90%), while two studies^23,24^ examined exposure across all trimesters. In addition, in one study^24^ the exposed cohort experienced only mild symptoms or were asymptomatic, while in another^23^ less than 5% experienced severe symptoms. In comparison, one study^21^ had a higher proportion of severe cases (~14%) than the others, and one study^22^ reported only moderate disease exposure. Exposure severity and timing of exposure in pregnancy are both considered important predictors of developmental outcomes,^26,27^ as the foetal brain undergoes rapid and complex biological changes at critical points in development and therefore may be at greater risk from environmental adversity at different stages.^28^ It should be noted, however, that sensitivity analyses performed in two studies^23,24^ did not reveal an association between trimester or severity of exposure with developmental test scores. As such, future research is needed to clarify the possible impact of timing and severity of SARS-CoV-2 antenatal exposure on developmental outcomes.

Another consideration is the potential effect of mother-infant separation which resulted from quarantine policies implemented at some hospitals to prevent neonatal exposure to SARS-CoV-2. Early separation could have an adverse effect on neurodevelopment due to the lack of mother-infant bonding opportunities through skin-to-skin contact,^29^ early interaction,^30^ and breastfeeding.^31^ Mother-infant separation was only explored in one article included in our meta-analysis.^21^ Infants in the exposure cohort were separated from their mothers significantly longer than infants in the non-exposure cohort (median of 37.5 days and 19 days respectively) and accounted for most of the total effect of SARS-CoV-2 on gross motor scores at 3 months. However, the sample was from Wuhan, China, and may not be representative of wider practice. Other secondary effects of the COVID-19 pandemic, such as reduced socialisation,^32^ mask wearing,^33^ and increased maternal and/or household stress^30^ are all likely to affect early childhood development. In one study,^23^ risk of delay in any developmental domain was greater for infants born during the pandemic (independent of exposure status) when compared to a historical cohort assessed before the pandemic. However, we do not expect this will have affected our results substantially as only studies that used a contemporaneous non-exposed cohort were included in the review. Therefore, this takes account of the impact of lockdown policies in general on children’s development.

Our findings are similar to recent meta-analyses examining infants’ developmental outcomes following antenatal exposure to maternal SARS-CoV-2 infection. Broadly, previous meta-analyses have suggested antenatal exposure was not related to an increased risk of delay in most domains of development, namely communication, gross motor, and personal social. One meta-analysis^18^ found no increased risk of delay for any domain, though did find exposed infants had lower developmental test scores for fine motor and problem-solving skills between 3 and 6 months than non-exposed infants. Another meta-analysis^16^ found that exposure related to a slightly increased risk of fine motor delay in infants assessed between ages 6 and 12 months. Thus, differences in the age at which development assessment occurred may account for the lack of evidence of risk of delay associated with antenatal exposure to maternal SARS-CoV-2 infection as the effects of exposure may be short lived or more reliably detected in later childhood. Indeed, one article reviewed in our meta-analysis^22^ reported that the five infants observed with fine motor delay in early infancy (~5 months of age) later showed improvements with parent-guided postnatal training and intervention. By 13 months of age all infants had subsequently passed the developmental assessment which indicates that developmental delay following SARS-CoV-2 maternal infection exposure may have minimal long-term impact or could be mitigated with appropriate early intervention. As such, ongoing observation and intervention of infants at risk for developmental delay is an important avenue for future research.

Motor delay in early infancy is linked to a greater risk of delay later in childhood^34^ and may also be predictive of later cognitive ability, such as working memory and processing speed, in school aged children.^35^ Motor and cognitive development are highly interrelated and brain regions responsible for motor and cognitive activity have a close functional relationship in early development.^36^ Therefore, it is important to investigate the potential impact of delayed motor and cognitive development observed in some studies in infancy associated with antenatal SARS-CoV-2 maternal infection exposure later in childhood.

### Neonatal exposure

Neurological assessment of newborn infants revealed exposure to the SARS-CoV-2 infection in the neonatal period was associated with slightly lower reflex and orientation behaviour, which is suggestive of impaired functioning of the central nervous system. However, developmental outcomes assessed using the ASQ-3 at 9 months of age indicated development was normal in all domains. Given the small sample of five participants and the young age at which development was assessed, current evidence on long-term neurodevelopmental outcomes of infants with exposure to the SARS-CoV-2 infection in the neonatal period is highly uncertain and future studies are needed.

### Strengths and limitations

A novel aspect of this review is that we examined developmental outcomes separately for term- and preterm-born infants; this is important given the well-established association between prematurity and poorer neurological outcomes. Yet, we encountered challenges as some data relating to preterm birth status was unavailable in the original research articles. Where we were unable to obtain the missing data from the authors, we assumed that the proportion of missingness was roughly the same for preterm and term-born infants. However, sensitivity analyses suggest that this assumption, even if incorrect, will have had little impact on the pooled estimates. In addition, our finding of no association between developmental outcomes and SARS-CoV-2 exposure in preterm born infants and SARS-CoV-2 exposure during the neonatal period is limited by the extremely small sample size. Further longer-term investigation is warranted to understand the potential developmental impact of SARS-CoV-2 infection exposure in these populations.

Another limitation is that two articles^23,24^ provided us with neurodevelopment assessment of some, but not all, of the same infants as a result of data sharing across multiple research groups. This means our sample was not completely independent and our meta-analysis findings may be unduly weighted by the outcomes of these infants. Most articles^21,23,24^ also utilised adjusted models to account for potential confounders and effect modifiers such as infant sex, infant age at assessment, maternal ethnicity, maternal age at delivery, and maternal educational levels in their analysis. While effect estimates in the unadjusted/minimally adjusted and fully adjusted models were similar in all cases, our meta-analysis results are unadjusted and thus may lack precision.

Our strict eligibility criteria allowed for a relatively homogeneous comparison of important study characteristics such as the study population, design, and outcome measures; however, this greatly reduced the number of studies included in our meta-analysis. We further found that the age at time of assessment and exposure timing and severity differed considerably across studies. Taken together, this has reduced our ability to understand the relatively disparate findings we have observed. Our review is further limited by the parental report nature of the ASQ-3 which is difficult to harmonise with findings of assessments conducted by healthcare professionals (DDST and DAYC).

Finally, given the young age at which development was assessed, our understanding of the long-term impact of antenatal or neonatal exposure to SARS-CoV-2 later in childhood remains unknown, as early developmental assessment has limited predictive value^37,38^ and developmental delays are more commonly and more reliably identified in older children.^39^ Large prospective longitudinal studies which measure development later in childhood are necessary to overcome this limitation.

## Conclusion

In this review we have identified no consistent association between antenatal and neonatal exposure to SARS-CoV-2 infection and developmental outcomes for term or preterm born infants below the age of 12 months. Continued research and monitoring of young children with early exposure to SARS-CoV-2 is needed to determine whether there are potential impacts of exposure on longer-term developmental outcomes.

### Supplementary information

Supplementary Materials

PRISMA 2020 Checklist

## Data Availability

All data generated or analysed during this study are included in this published article.
