# Supplementary material for: Antenatal and neonatal exposure to SARS-CoV-2 and children’s development: a systematic review and meta-analysis
Source: Pediatr Res. 2023 Dec 19;96(1):40–50. doi: 10.1038/s41390-023-02954-y (PMC11257989; doi:10.1038/s41390-023-02954-y)
Supplement: Supplementary file 1 — Supplementary Materials [file 41390_2023_2954_MOESM1_ESM.docx]

**Supplementary online information**

**Search terms**

Search terms identified in Table 1 were categorised under the key phrases “COVID-19”, “neurodevelopment”, and “fetus or neonate”. Search terms categorised under the same key phrase were combined using the OR Boolean operator (e.g., “COVID-19” OR “coronavirus” OR “coronavirus disease 2019” OR “SARS-CoV-2” OR “severe acute respiratory syndrome coronavirus 2”). Search term categorised under different key phrases were combined using the AND Boolean operator (e.g., (“COVID-19” OR “coronavirus” OR “coronavirus disease 2019” OR “SARS-CoV-2” OR “severe acute respiratory syndrome coronavirus 2”) AND (“Child development” OR “neurodevelopment” OR “neurobehaviour*” OR “neurobehavior*” OR “develop* adj3 (neuro* or motor or language or emotional)” OR “prenatal exposure delayed effects”). Some search terms included a truncation (indicated by an asterisk) to capture search terms with the same root but multiple endings. For example, the search term “neonat*” would capture the search terms “neonates” and “neonatal”. We also applied a proximity search term (indicated by operator “adj3”) which specified that one of the search terms within the following brackets following the “adj3” operator is located 3 words from the search term preceding the “adj3” operator. The search strategy was collaboratively developed by the authors and JH from the University Hospitals Bristol and Weston Library and Knowledge Services team and searches were undertaken by JH and KW.

Supplementary Table 1. Table of search terms used.

| **Key phrase** | **Search terms** |
| --- | --- |
| COVID-19 | COVID-19, coronavirus, coronavirus disease 2019, SARS-CoV-2, severe acute respiratory syndrome coronavirus 2 |
| Neurodevelopment | Child development, neurodevelopment, neurobehaviour* neurobehavior*, develop* adj3 (neuro* or motor or language or emotional), prenatal exposure delayed effects |
| Fetus or neonate | Infant, newborn, neonat*, Infan*, newborn*, fetus, foetus, fetal development, fetus development, maternal-fetal Exchange, fetomaternal transfusion, maternal exposure, prenatal exposure, in utero, maternal, pregnancy |
| * Indicates where truncation was used to capture all variations of the search term | |

Example of exact search terms used for database search
Ovid MEDLINE(R) ALL <1946 to May 03, 2023>

1 Infant, Newborn/ 666210
2 neonat*.mp. 336649
3 infan*.mp. 1413384
4 newborn*.mp. 832321
5 Fetus/ 82778
6 Fetus.mp. 141975
7 Foetus.mp. 9348
8 Fetal Development/ or fetus development/ 9482
9 Maternal-Fetal Exchange/ or fetomaternal transfusion/ 31266
10 Maternal Exposure/ or prenatal exposure/ 41327
11 in utero.mp. 31398
12 maternal.mp. 366309
13 Pregnancy/ 981639
14 1 or 2 or 3 or 4 or 5 or 6 or 7 or 8 or 9 or 10 or 11 or 12 or 13 2465275
15 COVID-19/ 221414
16 Coronavirus/ or coronavirus disease.mp. 74266
17 SARS-CoV-2/ or Severe acute respiratory syndrome coronavirus 2/ 152588
18 COVID-19.mp. 341922
19 Coronavirus.mp. 158445
20 SARS-CoV-2.mp. 200009
21 15 or 16 or 17 or 18 or 19 or 20 369488
22 14 and 21 14806
23 Prenatal Exposure Delayed Effects/ 33859
24 Child Development/ 50191
25 neurodevelopment.mp. 15123
26 neurobehaviour*.mp. 1774
27 neurobehavior*.mp. 14788
28 (develop* adj3 (neuro* or motor or language or emotional)).mp. 111361
29 23 or 24 or 25 or 26 or 27 or 28 209612
30 22 and 29 218
31 limit 30 to yr="2022 -Current" 117
32 limit 31 to dc=20221031-20230504 0
33 Infant, Newborn/ 666210
34 neonat*.mp. 336649
35 infan*.mp. 1413384
36 newborn*.mp. 832321
37 Fetus/ 82778
38 Fetus.mp. 141975
39 Foetus.mp. 9348
40 Fetal Development/ or fetus development/ 9482
41 Maternal-Fetal Exchange/ or fetomaternal transfusion/ 31266
42 Maternal Exposure/ or prenatal exposure/ 41327
43 in utero.mp. 31398
44 maternal.mp. 366309
45 Pregnancy/ 981639
46 33 or 34 or 35 or 36 or 37 or 38 or 39 or 40 or 41 or 42 or 43 or 44 or 45 2465275
47 COVID-19/ 221414
48 Coronavirus/ or coronavirus disease.mp. 74266
49 SARS-CoV-2/ or Severe acute respiratory syndrome coronavirus 2/ 152588
50 COVID-19.mp. 341922
51 Coronavirus.mp. 158445
52 SARS-CoV-2.mp. 200009
53 47 or 48 or 49 or 50 or 51 or 52 369488
54 46 and 53 14806
55 Prenatal Exposure Delayed Effects/ 33859
56 Child Development/ 50191
57 neurodevelopment.mp. 15123
58 neurobehaviour*.mp. 1774
59 neurobehavior*.mp. 14788
60 (develop* adj3 (neuro* or motor or language or emotional)).mp. 111361
61 55 or 56 or 57 or 58 or 59 or 60 209612
62 54 and 61 218
63 limit 62 to yr="2022 -Current" 117
64 limit 63 to dt=20221031-20230504 39

**Missing data**

In one study (Wu et al.), the article reported that 21 individuals (5 in the exposed group and 16 in the non-exposed group) had missing ASQ-3 data. However, the data provided to us by term/preterm status only gave means, standard deviations and numbers with developmental delay in each domain. The authors also provided the overall numbers exposed and non-exposed by preterm/term status, but did not provide information on numbers with missing data by preterm/term status. For the main analysis, we made the assumption that the proportions with missing data within the exposed and non-exposed groups were approximately the same for term and preterm infants, as shown below. In another study (Liu et al.), the article reported that five exposed infants and one non-exposed infant had fine motor delay; the article reported details, including gestational age, for the five exposed infants but not the non-exposed one. In the main analysis (to be conservative), this infant was included in both comparisons (term and preterm). We carried out sensitivity analyses making different assumptions about the missing data and re-calculated the pooled estimates. Details are given in Supplementary Table 2.

**Availability of data**

Original extracted data and data requested from authors are available publicly via the University of Bristol’s research data service: data.bris research data repository titled ‘SINEPOST Systematic review and meta-analysis’.

Supplementary Table 2. Pooled estimates obtained making different assumptions about missing data (Wu et al.; Liu et al.))

| Outcome | Main analysis:^1^ | Wu et al: assuming all those with missing data were term-born^2^ | Wu et al.: assuming the maximum possible number with missing data were preterm^3^ |
| --- | --- | --- | --- |
| Term-born infants | | | |
| Standardised mean difference:  Communication  Gross motor  Fine motor  Problem solving  Personal social | 0.03 (-0.18, 0.24)  -0.18 (-0.63, 0.28)  0.04 (-0.44, 0.52)  -0.16 (-0.42, 0.11)  -0.34 (-1.15, 0.47) | 0.03 (-0.18, 0.24)  -0.18 (-0.63, 0.28)  0.04 (-0.44, 0.52)  -0.15 (-0.42, 0.11)  -0.34 (-1.15, 0.47) | 0.03 (-0.18, 0.24)  -0.18 (-0.63, 0.28)  0.03 (-0.45, 0.52)  -0.16 (-0.43, 0.11)  -0.34 (-1.15, 0.47) |
| Odds ratio:  Communication  Gross motor  Fine motor^4^  Problem solving  Personal social | 0.73 (0.24, 2.24)  1.50 (0.62, 3.62)  2.90 (0.58, 14.43)  1.19 (0.54, 2.66)  1.93 (0.78, 4.75) | 0.73 (0.24, 2.24)  1.50 (0.62, 3.62)  2.90 (0.58, 14.45)  1.19 (0.54, 2.67)  1.93 (0.79, 4.76) | 0.73 (0.24, 2.22)  1.50 (0.62, 3.61)  2.88 (0.58, 14.22)  1.18 (0.53, 2.63)  1.90 (0.77, 4.68) |
| Preterm infants | | | |
| Standardised mean difference:  Communication  Gross motor  Fine motor  Problem solving  Personal social | -0.17 (-0.69, 0.34)  0.04 (-0.44, 0.52)  -0.20 (-0.68, 0.27)  -0.27 (-0.74, 0.21)  0.35 (-0.37, 1.07) | -0.17 (-0.67, 0.33)  0.02 (-0.45, 0.49)  -0.21 (-0.68, 0.26)  -0.27 (-0.75, 0.20)  0.33 (-0.37, 1.02) | -0.17 (-0.71, 0.37)  0.08 (-0.41, 0.57)  -0.18 (-0.67, 0.30)  -0.24 (-0.73, 0.25)  0.40 (-0.36, 1.16) |
| Odds ratio:  Communication  Gross motor  Fine motor^5^  Problem solving  Personal social | 1.62 (0.24, 10.91)  0.53 (0.10, 2.73)  1.22 (0.27, 5.58)  1.16 (0.22, 5.99)  0.77 (0.10, 5.81) | 1.64 (0.25, 11.04)  0.53 (0.10, 2.76)  1.24 (0.27, 5.64)  1.18 (0.23, 6.07)  0.79 (0.11, 5.93) | 1.71 (0.25, 11.66)  0.54 (0.10, 2.83)  1.28 (0.28, 5.99)  1.23 (0.23, 6.52)  0.83 (0.10, 6.54) |

1. In the exposed cohort there were 57 infants (49 term-born and 8 preterm); 5 of these (9%) did not have ASQ-3 data. 9% of 49 is 4 (to nearest whole number), so in the main analysis we assumed 4 term infants and 1 preterm infant in the exposed group had missing ASQ-3 data. Similarly, in the non-exposed cohort there were 78 infants (73 term, 5 preterm); overall, 16 (21%) had missing ASQ-3 data. 21% of 73 is 15, so in the main analysis we assumed 15 term infants and 1 preterm infant had missing ASQ-3 data.
2. In this analysis we assumed that all 5 exposed infants and all 16 non-exposed infants with missing ASQ-3 data were term-born.
3. In this analysis we assumed that all 5 exposed infants with missing ASQ-3 data were preterm and none term-born and, among the 16 non-exposed infants with missing ASQ-3 data, 3 were preterm (since we were given standard deviations for the scores in each domain, there must have been at least 2 infants in this group) and 13 were term-born.
4. If the non-exposed infant with fine motor delay was not actually term-born, the odds ratio (95% CI) for the main analysis would have been 3.05 (0.46, 20.30).
5. If the non-exposed infant with fine motor delay was not actually preterm, the odds ratio (95% CI) for the main analysis would have been 1.58 (0.33, 7.58).
